# Unbreakable in Crisis: A Systematic Review Exploring Nurse Resilience and Contributing Factors During the COVID‐19 Pandemic

**DOI:** 10.1002/puh2.70015

**Published:** 2025-02-21

**Authors:** Jibin Kunjavara, Rinu J. George, Manoj Kumar L., Shiny T. Sam, Kamaruddeen Mannethodi

**Affiliations:** ^1^ Hamad Medical Corporation Doha Qatar; ^2^ TMM College of Nursing Thiruvalla India; ^3^ NHS Nottingham UK

**Keywords:** burnout, coronavirus disease 2019 (COVID‐19), frontline, nurses, psychological, resilience

## Abstract

The coronavirus disease 2019 (COVID‐19) placed an unprecedented burden on the global healthcare system, severely affecting the physical and mental health of healthcare workers, particularly nurses. Nurses faced immense workloads, increased infection risks, uncertainty, and public scrutiny. Despite these challenges, nurses were lauded for their dedication and resilience in confronting the pandemic. Many experienced mortality, morbidity, and post‐COVID sequelae. This review integrates psychological resilience literature from 2020 to 2022, utilizing Whittemore and Knafl's integrative review method. A total of 22 studies met the inclusion criteria, focusing on how nurses demonstrated resilience during the COVID‐19 crisis. Most studies reported a moderate level of resilience, with a mean score of 62.54. Factors such as positive acceptance of change, trust in personal judgment, perceived competence, and spiritual influences were positively associated with resilience, whereas burnout, anxiety, and depressive thoughts negatively impacted it. Resilience was found to mediate the relationships between variables like perceived stress, emotional exhaustion, and quality of life. The literature suggests that healthcare administrations should foster a healthy work environment, maintain an optimistic outlook, and establish strong connections with frontline staff to mitigate the pandemic's impact. Providing supportive environments, resilience training, and mental health interventions will be crucial in enhancing resilience for future crises.

## Graphical abstract

This review highlights the resilience of nurses during the COVID‐19 pandemic, exploring factors that influenced resilience, such as positive acceptance of change, competence, and spiritual influences. The graphical abstract illustrates the relationships between key psychological variables, showing resilience as a mediator between stress, emotional exhaustion, and quality of life. A supportive work environment and mental health interventions are emphasized as crucial strategies for enhancing resilience in preparation for future crises.

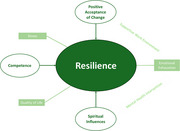

## 1 Introduction/Background

The coronavirus disease 2019 (COVID‐19) has significantly strained healthcare systems worldwide. Nurses, the largest segment of the healthcare workforce, faced heightened risks due to excessive workloads, resource shortages, and the constant fear of infection and stigmatization [1]. Pandemics and other crises naturally elevate stress and anxiety levels among healthcare workers. Clinicians around the globe face issues, including high mortality of patients in their care, high healthcare demands, rationing of healthcare supplies, and extraordinary physical and emotional stress [2]. Similarly, the pandemic negatively affects the nurse workforce, the largest professional category in the healthcare sector. Additionally, to deal with the enormous workload with inadequate resources, nurses put themselves and their loved ones’ life at risk to accomplish their responsibilities [3].

Nurses are at increased risk of developing psychological problems under these stressful work situations. During the pandemic, nurses were working under extreme physical and emotional pressure while dealing with patients, which could further amplify their anxiety and fears. A study in Spain revealed that one in seven healthcare workers suffer from psychiatric disorders, such as major depressive disorder, anxiety disorders, panic attacks, and post‐traumatic stress disorder early in this pandemic [4]. Additional stressors such as resource shortages, ethical dilemmas, and extended hours in high‐risk settings exacerbated the psychological toll [5]. Moreover, many nurses were deployed to COVID isolation facilities or areas outside their usual clinical practice or expertise, often working long hours according to the demand of the clinical site or higher admission rate of patients. The healthcare workers were highly distressed and concerned about transmitting COVID‐19 to family and loved ones [6]. The widespread shortage of personal protective equipment (PPE), lack of testing equipment, and uncertainty surrounding COVID‐19 treatment protocols further compounded nurses’ distress [7].

Resilience is a process of successful adaptation through hardships or significant sources of stress [8]. Resilience allows nurses to maintain their mental and psychological health in times of crisis. During the COVID‐19 pandemic, negative emotions such as companionate fatigue, discomfort, and helplessness were dominant in the early stages of the pandemic. Later, it was shown that the growth of positive emotions, such as increased affection and gratitude, and the development of professional responsibility coexisted over time [9]. Despite this, the factors influencing nurses’ ability to cope with stress, anxiety, or depression among healthcare workers during the COVID‐19 pandemic have not been thoroughly examined [10]. Additionally, nurses are facing problems in their everyday work environment such as acute shortages of nurses, lack of retention of trained nurses, higher patient–nurse ratio, implementation of new technology, regulatory requirements to maintain their professional license, physical and psychological demands of the work, and ethical dilemmas.

A study found that nurses in China showed a spirit of “resilience within the challenge” to overcome the COVID‐19 crisis. However, little is known concerning the resilience level and contributing factors of nurses during the COVID‐19 pandemic. In this review, the authors provide current knowledge on the resilience level and contributing factors for nurses during the COVID‐19 pandemic. For the formulation of successful organizational strategies to better support the resilience of nurses, a broader understanding of the topic is essential [11].

This review seeks to synthesize and integrate current knowledge on nurse resilience during the pandemic. Understanding the factors that promote or hinder resilience is essential for developing organizational strategies that support nurses. The findings of this review hold implications for research, practice, and policy. By identifying resilience‐building strategies, nursing leaders can enhance training, improve retention, and ensure that frontline nurses are better equipped to manage future healthcare crises. Policymakers should consider resilience a key component of healthcare workforce well‐being and integrate it into guidelines to strengthen healthcare system sustainability in the face of adversity.

### 1.1 Aim and Objectives

The review was aimed at understanding the nurse's resilience level during the COVID‐19 pandemic and the contributing factors to nurses resilience during the pandemic.

## 2 Methods

This review followed Whittemore and Knafl's integrative review method. Whittemore and Knafl described integrative review methods as the most comprehensive and updated review method because it permits reviewers to include various articles, including qualitative and empirical quantitative studies and theoretical reports [12]. So, this method was applied to minimize the review bias and increase the accuracy of the findings. Whittemore and Knafl recognized six phases of their review method: problem identification, literature search, data evaluation, data analysis, and presentation of conclusions.

### 2.1 Literature Search

In accordance with Whittemore and Knafl's method, the objective of the literature search phase is to incorporate as many suitable primary sources as possible within a predefined frame [9]. For this review, the literature search was conducted using a preplanned search strategy to identify current articles. Initially, six investigators independently searched the literature using the electronic databases CINAHL, PubMed, Cochrane, Joanna Briggs Institute, OVID EMCARE, ERIC, Scopus, and ProQuest.

To broaden the search and identify other gray literature, Google and advanced Google Scholar were searched. In addition, the reference lists and citations of all relevant studies were hand‐searched for further pertinent literature. In the search strategy, a combination of search terms and keywords using Boolean operators, phrase searching, truncation, and Medical Subject Headings (MeSH) was used. The keywords used in this search were “Nurse,” “Resilience,” “COVID‐19,” OR “Pandemic.” During the manual, title/abstract, and full‐text screening, the included articles will be classified according to these terms. The limitation of the search includes articles in English, the years between 2020 and 2022, to focus the review on the most recent literature peer‐reviewed articles only.

**FIGURE 1:**
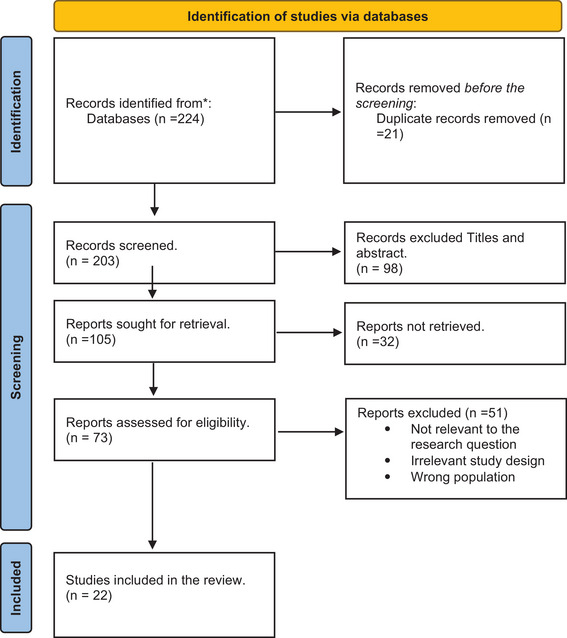
PRISMA flowchart.

The steps of the literature search are presented in the Preferred Reporting Items for Systematic Reviews and Meta‐Analyses (PRISMA) flowchart (Figure 1). The initial database search yielded 224 articles. Limiting the search to the years between 2020 and 2022 focused the review on the most recent literature, resulting in 105 articles. When the search was additionally limited to academic journals only, the number of articles became 98. Finally, 90 articles remained when the search was limited to English‐only articles. The authors reviewed the titles and abstracts of articles and identified 46 relevant articles that met the inclusion criteria. After reviewing the full text, 19 were selected. In the supplemental search of Google Scholar, three more articles were included in the review. A total of 22 articles were included in this review.

### 2.2 Inclusion Criteria

Only studies reporting the nurse's resilience level and their contributing factors during COVID‐19 were selected. The study population included in this review is nurses who are working during COVID‐19.

### 2.3 Exclusion Criteria

Non‐research articles and studies not including nurses were excluded from the review. Articles that discussed resilience in other professions but did not include nurses were excluded. For example, articles that examined resilience among physicians were excluded; however, articles that examined resilience among physicians and nurses were included, and data regarding nursing were included in the analysis and result.

The selected articles were reviewed by the independent reviewer to ensure consistency with the application of inclusion and exclusion. The steps of the literature search are presented.

### 2.4 Data Evaluation

Quality assessment was performed by two reviewers (S.P., P.S.) who independently appraised the methodological quality of the included studies using a modified Johns Hopkins Nursing Evidence‐Based Practice Research Evidence Appraisal Tool [13]. A one‐point score was assigned to each of the 12 appraisal items, and the quality score ranged from 12 to 0. The score was divided into three levels as follows: high quality (score between 12 and 9), good quality (score between 8 and 5), and low quality (score between 4 and 0). Most of the articles (18 articles) obtained a score above nine, and four articles had a score of 8 which was considered good quality. The appraisal report was reviewed and discussed by other members of the research team, and they reached a consensus on the score. The quality assessment is summarized in Table 1.

**TABLE 1:** Modified Johns Hopkins nursing evidence‐based practice research evidence appraisal tool.

Sl no.	Appraisal items	Yes (number of articles)	No
1	Does the researcher identify what is known and not known about the problem and how the study will address any gaps in knowledge?	18	4
2	Was the purpose of the study clearly presented?	19	3
3	Was the sample size sufficient based on the study design and rationale?	20	2
4	Was the literature review current (most sources within the last 5 years or classic)?	18	4
5	Are data collection methods described clearly?	22	0
6	Were the instruments reliable (Cronbach's *α* [alpha] > 0.70)?	15	7
7	Was instrument validity discussed?	17	5
8.	If surveys/questionnaires were used, was the response rate >25%?	19	3
9.	Were the results presented clearly?	20	2
10.	If tables were presented, was the narrative consistent with the table content?	22	0
11.	Were study limitations identified and addressed?	21	1
12	Were conclusions based on results?	22	0

### 2.5 Data Extraction

The data were extracted from primary sources and organized into a comprehensible structure, such as a matrix or spreadsheet. The data were compiled into a presentation on the basis of the certain factors or categories [14]. Data extracted, including author and year, nationality (post‐COVID‐19), method, resilience, and contributing factors to develop the resilience recommended to develop resilience among nurses, were extracted using a spreadsheet (Table 2).

**TABLE 2:** Description of the included studies.

Number	Reference	Design, sample, and setting	Result	Appraisal score
1	[15]	Descriptive cross‐sectional research study 120 frontline nurses in emergency care areas in India	Showed a moderate to high level of resilience (77.77 ± 12.41). Emotional exhaustion and personal inefficacy had a significantly negative correlation with resilience among the frontline nurses in the emergency (*r* = 0.25, *p* < 0.05 and *r* = 0.31, *p* < 0.01, respectively). A significant negative correlation has been identified between burnout and resilience that informs the role of resilience in alleviating burnout during this pandemic	10
2.	[16]	A cross‐sectional study 196 healthcare workers (doctors and nurses) in Portugal	Showed moderate resilience (64.55 ± 7.47) Emotional exhaustion: −0.17; 95% confidence interval (95% CI): −0.38, 0.04; depersonalization: −0.17; 95% CI: −0.31, −0.03; personal achievement: 0.50; 95% CI: 0.40, 0.61 Emotional exhaustion↑ Pandemic situation↑	8
3.	[17]	Cross‐sectional research study 202 Iranian nurses in educational hospitals	The result shows a moderate level of resilience (61.45 ± 6.42) The quality of work‐life also had negative and significant effects on emotional exhaustion (*p* < 0.001, *β* = −0.38) and reduced personal accomplishment (*p* < 0.001, *β* = −0.38)	9
4	[18]	Cross‐sectional correlational design 550 registered Nurses Hospitals in Jordan	The mean score for perceived stress was 19.50, and the mean score for resilience was 61.57 The results indicated that resilience was negatively correlated with perceived stress and quality of life. Further, resilience was found to play a partial mediating role in the relationship between perceived stress and quality of life	11
5	[19]	This research is a cross‐sectional study 100 nurses Large Hospital in Tehran, Iran	The mean scores of resilience were 72.38 ± 7.11 “Positive acceptance of change,” “spiritual effects,” and “trust in individual instincts,” “perception of competence,” “number of patients monitored in each work shift,” “number of shifts per month,” “level of education,” and “gender”	10
6	[20]	Designed as a cross‐sectional study using a questionnaire to gather demographic data of the nurses, they included 92 nurses by convenient sampling, starting from the month of February 2020, China	The total resilience score was 87.04 ± 22.78. The SCL‐90 score was 160–281 (202.5 ± 40.79). Somatization, obsessive‐compulsive, anxiety, phobic anxiety, and psychoticism domains and scores were like national averages (*p *> 0.3). Apart from somatization and other domains, the mean resilience score was negatively associated with the scores of other SCL‐90 domains	11
7	[14]	A cross‐sectional study was performed using an online questionnaire distributed via social. A total of 2008 subjects completed the survey in Portugal	Participants showed a moderate level (50.8%) or high level of psychological resilience (27.8%) The results revealed that depression had not only a directed effect on personal, work‐ and client‐related burnout but also an indirect small effect on it through resilience. Psychological resilience played a partial mediating role between depression and all burnout dimensions	9
8	[21]	A cross‐sectional survey was conducted from January to March 2021 in seven long‐term care hospitals in the Seoul metropolitan area to measure resilience, nursing professionalism, and job stress among nurses	Participants showed a moderate level of 62.2 ± 12.2 Nursing professionalism had a significant mediating effect on the relationship between resilience and job stress levels. The effect of resilience on job stress levels was significant (*β* = −0.16, *p* = 0.024)	11
9	[22]	A cross‐sectional approach and correlational design. A total of 180 front‐line nurses were assigned to the task force in Wuhan City between March and April 2020, China	The total prevalence of burnout was 51.7%, of which 15.0% was severe burnout. These preliminary results revealed that positive and negative affect fully mediated the effects of resilience on burnout, emotional exhaustion, depersonalization, and reduced personal accomplishment of front‐line nurses	10
10	[23]	A cross‐sectional and correlational study was conducted with 284 nurses in Turkey Not given the rate of resilience	These values suggest that the participants had a moderate level of psychological resilience Psychological resilience explained 5% of job performance. The psychological resilience levels of the nurses who were ≥41 years old and who did their job enthusiastically were higher In other words, a one‐unit increase in nurses’ psychological resilience levels will increase their job performance levels by 0.05 units	8
11	[24]	Cross‐sectional The study was conducted with 418 physicians and nurses between July and August 2020, in Turkey	The psychological resilience level of the physicians (18.42 ± 2.25) participating in the study was higher than that of the nurses (17.88 ± 2.00). Both physicians and nurses who stated that the hospital environment negatively affected their psychological health, that their motivation to work decreased, that they were more tense, angry, and intolerant in daily life, and that they felt physically weak were found to have low psychological resilience and high perceived stress levels	9
12	[25]	Cross‐sectional study The respondents were healthcare workers at Dr. Soetomo Hospital as the COVID‐19 referral hospital in Surabaya, East Java, Indonesia. Data were collected from June 10 to June 16, 2020. 227 respondents filled out the questionnaire online	The mean score of the respondents’ resilience was 69 ± 15.823. The Spearman correlation test showed a significant relationship between anxiety and resilience. The direction of the relationship between anxiety and the total resilience score is negative with moderate relationship strength. This means that the higher the anxiety, the lower the total resilience score	10
13	[26]	Cross‐sectional and quantitative analyses. Survey data were obtained during the period between April 1 and May 25, 2020 in a sample of 421 nurses from 39 Spanish provinces	(a) All the stressors have a significant, direct, and negative relationship with nurses’ psychological distress; (b) emotion‐focused strategies are negatively related to nurses’ psychological distress directly and indirectly through resilience, and (c) problem‐focused strategies are positively related to nurses’ psychological distress and negatively and indirectly through emotion‐focused strategies	11
14	[27]	Qualitative research design In‐depth interviews were conducted among 21 participants from March to May 2020, China	They displayed an ability to bounce back from negative mental experiences and transform into a positive mindset to cope with the stress they faced. Factors that enhanced the nurses’ resilience during the pandemic were, they are becoming familiar with infectious disease protocols, having a sense of professional achievement, receiving social support, having trust in the infection‐control response team in the hospital, and using self‐regulation strategies	8
15	[28]	Qualitative research design Experiences of 18 Israeli nurses who are directly treating COVID‐19 patients	The analysis yielded three central analytic themes that described the nurses’ experiences during the pandemic: maneuvering between professional demands and personal‐family life; the nurses’ coping strategies and resilience; and nurses' use of metaphorical military language as a way of coping with the difficulties. The findings show that in a time of severe health crisis, and despite the fear of infection, nurses adhere to the values of the profession and are willing to fight the virus to save lives	9
16	[29]	The study was descriptive and cross‐sectional the study comprised 720 nurses working at a university hospital in an eastern province in Turkey. The sample was composed of 370 voluntary nurses working at the relevant hospital between April 12 and 17, 2020	The total average CD‐RISC score was 64.28 ± 15.99. On the basis of the subdimension and total average MSPSS scores of the nurses. Factors that contributed to resilience were spiritual tendency, tolerance of negative effects, competence, tenacity, and personal	11
17	[30]	This cross‐sectional study included 377 midwives and nurses in Turkey	The mean psychological resilience score was significantly lower in midwives and nurses with high depression scores (*p* < 0.001). The mean scores for emotional exhaustion and depersonalization were significantly higher in midwives and nurses with high depression scores, whereas the personal accomplishment score was significantly lower in this group. According to the burnout subscales, burnout, depersonalization, and low personal achievement were significant in midwives and nurses who had high depression scores (*p* < 0.01)	9
18	[31]	Cross‐sectional survey We extracted data from 824 nursing professionals, China	The mean score of the respondents’ resilience was 58 ± 15.823 PHQ‐9 score positively correlated with high GAD‐7, SAVE‐6, and SAVE3 scores and low BRS scores. BRS score negatively correlated with PHQ‐9, GAD‐7, SAVE‐6, and SAVE‐3 scores	10
19	[32]	Cross‐sectional Self‐report scales were used to collect data from 270 frontline nurses in selected hospitals in the Philippines	Overall, 38.5% of frontline nurses experienced medium to high CF during the second wave of the pandemic. Increased CF was associated with poorer nurse‐reported quality of care (*β* = −0.145, *p* = 0.019), lower job satisfaction (*β* = −0.317, *p* = 0.001), and higher organizational turnover intention (*β* = 0.301, *p* = 0.001). Moreover, resilience fully mediated the relationship between CF and quality of care (*β* = −0.088, *p* = 0.169), and partially mediated the relationship between CF and job satisfaction (*β* = −0.259, *p* = 0.001), and CF fatigue and organizational turnover intention (*β* = 0.272, *p* = 0.001)	12
20	[33]	A Cross‐sectional survey designs. The sample consisted of 152 frontline healthcare workers, physicians, and paramedical professionals including nurses in Italy	The mean psychological resilience score was significantly higher in healthcare workers. The mean score was 67 ± 11.4 The results are shown in Table 1 and confirmed significantly higher levels of stress and anxiety in females, whereas differences were found related to neither the professional role nor to the interaction of the role with the gender. The self‐reports of stress and anxiety are significantly correlated with each other (*r* = 0.58, *p* < 0.01); the correlations with the other variables considered in the study (personality factors, resilience, job seniority)	8
21	[34]	The study was carried out as a case–control study with the participation of 400 nurses as the target group (nurses exposed to COVID‐19 patients) and the control group (nurses not exposed to COVID‐19 patients) Iran	The mean scores of job stress and resilience were 190.59 ± 22.56 and 61.13±13, respectively The mean scores for job stress and resilience were significantly different between the target and control groups (*p* < 0.05). Theo and resilience in the target group were less than that in the control group. In addition, job stress in the target group was higher than that of the control group (*p* < 0.05). There was a significant and negative correlation between resilience and job stress, and the correlation was stronger in the target group (*p* < 0.05)	9
22	[35]	Qualitative study In‐depth interviews were conducted with PCPs (*n* = 19), who served at COVID‐19 facilities in India	Lack of epidemic management and intensive tertiary care experience, limited and inadequate training, and fear of infection emerged as the primary sources of distress, in addition to the absence of systemic mental health support and formalized recognition	10

Abbreviation: COVID‐19, coronavirus disease 2019.

### 2.6 Included Studies

The initial database search yielded 224 articles. It limited the search to the years between 2020 and 2022 to focus the review on the most recent literature resulting in 105 articles. When the search was additionally limited to academic journals only, the number of articles became 98. Finally, 90 articles remained when the search was limited to English‐only articles. The author reviewed the titles and abstracts of articles and identified 46 relevant articles that met the inclusion criteria (Table 1). After reviewing the full text, 19 were selected. In the supplemental search of Google Scholar, three more articles were included in the review. A total of 22 articles were included in this review. Table 2 provides a full description of the included studies.

## 3 Results

The included studies reported a wide range of psychological variables regarding resilience. Most of the studies were cross‐sectional (*n* = 18), three were qualitative designs, and one study was case–control. Most included studies explained resilience rates and factors contributing to psychological resilience. Many of the studies included have a moderate level of resilience (mean score = 62.54), whereas one study reported a low level of resilience (mean score = 18.42). Ebrahimi et al. report that the nurses had a moderate level of resilience (mean score = 72.38) and the factors that contributed to their resilience include positive acceptance of change, spiritual effects, trust in individual instincts, perception of competence, number of patients monitored in each work shift, number of shifts per month, and level of education [19].

The studies also revealed a significant negative correlation between emotional exhaustion and personal inefficacy with resilience among frontline nurses in emergency departments. Strength was found to mediate the relationship between exhaustion, perceived stress, and quality of life [10, 11, 15–17]. A cross‐sectional study carried out in Jordan explained that resilience was negatively correlated to perceived stress and quality of life. Emotion‐focused strategies were negatively related to nurses' psychological distress directly and indirectly through strength [21, 25].

Additionally, somatization, obsessive‐compulsive, anxiety, phobic anxiety, and psychoticism domains and scores were like national averages (*p* > 0.3). Apart from somatization and other fields, the mean resilience score was negatively associated with the anxiety scores SCL‐90 domains. The psychological resilience levels of nurses were high among those who were enthusiastic and aged over 41 years [14, 18, 25]. Boran et al. identified that almost all the participants (99.3%) were afraid of transmitting COVID‐19 to their families [24]. The physician's psychological resilience was higher (M = 18.42, SD = 2.25) compared to nurses (M = 17.88, SD = 2.00). Both physicians and nurses stated that the hospital environment negatively affected their psychological health, which included lack of motivation to work, experiencing tension, angry, intolerant in daily life, physically weak, low psychological resilience, and high perceived stress levels [24]. The factors that contribute to resilience among nurses include spiritual tendency, tolerance of adverse effect, competency, and tenacity [25].

In addition to that, the three studies explore the resilience level of nurses by qualitative approach, and it is produced by several themes like experiences during the pandemic: maneuvering between professional demands and personal‐family life, the nurses’ coping strategies, and resilience. Nurses use metaphorical military language to cope with difficulties. The findings show that in a time of crisis, despite the fear of infection, nurses adhere to the values of the profession and are willing to fight the virus to save the lives of their patients. More than that, they displayed an ability to bounce back from negative mental experiences and transform into a positive mindset to cope with the stress they faced. Factors that enhanced the nurses’ resilience during the pandemic were that they are becoming familiar with infectious disease protocols, having a sense of professional achievement, receiving social support, having trust in the infection‐control response team in the hospital, and using self‐regulation strategies. Some of the obstacles they pointed out to achieving resilience were lack of epidemic management, lack of intensive tertiary care experience, limited and inadequate training, and fear of infection, in addition to the absence of systemic mental health support and formalized recognition.

## 4 Discussion

The primary objective of this review was to evaluate the level of nurse resilience during the COVID‐19 outbreak and explore how resilience is developed and maintained in challenging healthcare environments. The review highlights the critical role resilience plays in enabling healthcare professionals, particularly nurses, to cope with the psychological challenges posed by the pandemic. Healthcare professionals are forced to work under challenging conditions owing to the COVID‐19 virus outbreak. Under such circumstances, many essential healthcare workers become psychologically traumatized and require psychological support [36].

The findings from the included studies indicate that resilience is inversely related to emotional exhaustion and personal inefficacy, especially among emergency nurses. This underscores the need for healthcare administrations to foster a supportive work environment. Ensuring a healthy atmosphere and promoting positive relationships among frontline workers can mitigate the detrimental effects of the pandemic on mental health. Previous literature [11] supports this, demonstrating that resilience is crucial in protecting against burnout, with work‐life balance serving as an essential factor in reducing emotional fatigue. The nursing managers can promote the nurses’ resilience and reduce burnout by using the health service workplace environmental resilience model (HSWERM) [20]. Depression and moderate‐to‐severe anxiety disorders were reported among many nurses. The depression group was more concerned with motivation, escape from work, loneliness and unjust treatment, anxiety, fear of infection, death, and isolation compared with the non‐depression group. These emotional states can be alleviated through targeted interventions such as mental health support, rapid online counseling services, and resilience‐building training programs.

Occupation variables significantly predicted healthcare workers’ psychological resilience. Nurses who tested positive for COVID‐19 reported lower resilience levels, suggesting that personal health threats, despite safety precautions, can diminish psychological resilience [36]. In addition to that, nurses who tested positive for COVID‐19 reported lower resilience than those who reported negative. This may be because the positive COVID‐19 statuses, despite safety precautions, threatened their sense of resilience [37].

Extensive research is needed to determine the exact factors and interventions that promote psychological resilience. Early mental distress diagnosis and interventions are needed to build a healthy clinical workforce for COVID‐19 treatment and control. Increased mental health financing is needed, especially for clinicians who identify psychological distress (depression, anxiety) [38]. Interventions and policy changes are required to increase long‐term care nurses’ resilience and professionalism throughout the COVID‐19 epidemic [39]. Immediate insights give research‐based resilience techniques that manage burnout through positive and negative consequences in HCWs to promote well‐being during pandemics.

Nurses’ resilience is a vital component of their emotional work of coping with patients’ illnesses and death. In conclusion, resilience plays a vital role in helping nurses manage the emotional demands of their profession, especially during global health crises. Without adequate resilience, nurses may face burnout, which could lead to attrition from the profession [40]. Therefore, it is imperative that healthcare institutions and governments prioritize the well‐being of their workforce by implementing strategies that build and maintain resilience, ultimately fostering a more sustainable and resilient healthcare system.

## 5 Limitation

Even though this study adopted an integrative review method that enabled the inclusion of studies with various designs, the lack of a statistical method, which can be utilized in meta‐analyses and would confirm the statistical significance of the impact of the COVID‐19 pandemic on psychological resilience, could be considered a limitation. So, future reviews in this area should implement meta‐analysis review methods that enable the use of statistical methods. Nevertheless, the finding of this review provides a comprehensive appraisal and integration of the current literature on psychological resilience during COVID‐19. Another limitation is that reviewers conducted this review from different places, which might impact the accuracy of the review. The primary author consulted a nursing research expert several times during the review to alleviate this limitation. Findings explain that a brief, online intervention can improve healthcare workers’ mental health during a crisis such as the COVID‐19 pandemic. There is a need to increase individual resilience and nursing professionalism through intervention programs and policy proposals to manage job stress among long‐term care hospital nurses for future pandemics.

## 6 Conclusion

This review highlights key factors that contribute to nurse resilience during the COVID‐19 pandemic, offering actionable insights for nursing administration. Positive acceptance of change, spiritual influences, trust in one's judgment, and perceptions of competence are shown to enhance resilience, whereas burnout and depressive thoughts undermine it. Nursing administrators must focus on strategies that foster mental well‐being and resilience, such as creating supportive environments and resilience training programs. These efforts can help nurses manage emotional challenges and improve their long‐term commitment to the organization. In short, healthcare organizations must prioritize resilience‐building strategies to support a sustainable and robust nursing workforce capable of facing future challenges.

## Author Contributions

**Jibin Kunjavara**: conceptualization, writing–original draft. **Rinu J. George**: methodology, writing–original draft. **Manoj Kumar L**.: conceptualization, writing–review and editing. **Shiny T. Sam**: conceptualization, writing–original draft. **Kamaruddeen Mannethodi**: methodology, writing–review and editing.

## Disclosure

The support provided by the Qatar National Library was instrumental in facilitating the comprehensive literature search and the overall completion of this integrative review.

## Ethics Statement

This integrative review was conducted in adherence to the ethical guidelines and standards for research. As this study did not involve human participants, animals, or any form of personal data, ethical approval from an Institutional Review Board (IRB) was not required. However, ethical principles were strictly followed to ensure the integrity and accuracy of the research. All sources of data were appropriately cited, and efforts were made to include a comprehensive range of studies to minimize bias and ensure the reliability of the findings.

## Conflicts of Interest

The authors declare no conflicts of interest.

## Data Availability

The data used and analyzed in this review are publicly available in the articles referenced within the manuscript. This review synthesizes findings from previously published studies, and no new data were generated. All relevant data are included in the manuscript and its supplementary materials. For additional details on the datasets analyzed, readers can refer to the individual studies cited.
